# The role of PI3K/Akt signal pathway in the protective effects of propofol on intestinal and lung injury induced by intestinal ischemia/reperfusion^1^

**DOI:** 10.1590/s0102-865020190010000005

**Published:** 2019-02-14

**Authors:** Qingwen Li, Shanshan Cui, Guoqing Jing, Huang Ding, Zhongyuan Xia, Xianghu He

**Affiliations:** I MD, Department of Anesthesiology, Renmin Hospital of Wuhan University, Hubei, China. Manuscript preparation and writng.; II MD, Department of Anesthesiology, Renmin Hospital of Wuhan University, Hubei, China. Conception and design of the study, acquisiton of data.; III MD, Department of Anesthesiology, Renmin Hospital of Wuhan University, Hubei, China. Analysis and interpretation of data, technical procedures.; IV MD, Department of Anesthesiology, Renmin Hospital of Wuhan University, Hubei, China. Histopathological examinations, statistics analysis.; V MD, Department of Anesthesiology, Renmin Hospital of Wuhan University, Hubei, China. Final approval.; VI MD, Department of Anesthesiology, Zhongnan Hospital of Wuhan University, Hubei, China. Critical revision.

**Keywords:** Intestine, Lung, Propofol, Ischemia, Reperfusion, Oxidative Stress, Apoptosis, Rats

## Abstract

**Purpose:**

To investigate the role of PI3k/Akt signal pathway in the protective effects of propofol on intestinal and lung injury induced by intestinal ischemia/reperfusion(I/R).

**Methods:**

Male Sprague-Dawley rats were subjected to 45 min of ischemia by occluding the superior mesenteric artery and to 2h of reperfusion to establish the model of I/R. Twenty four rats were randomly divided into four groups: Sham, intestinal I/R (II/R), propofol (P), wortmannin (W). In groups P, W, propofol was injected intravenously and continuously at the onset of reperfusion via infusion pump. PI3K inhibitor (wortmannin) was administered intravenously in group W 25 min before ischemia. Intestinal tissues and lung tissues were obtained for determination of histologic injury, wet/dry weight ratio, malondialdehyde (MDA) levels, superoxide dismutase (SOD) and myeloperoxidase (MPO) activities. Meanwhile, the expressions of caspase-3 and phosphorylated Akt (p-Akt) in intestines and lungs were detected by western blot.

**Results:**

Propofol treatment alleviated intestinal and lung morphological changes which were observed in II/R group , Moreover, wet/dry weight ratio, the MDA level, MPO activity and expression of caspase-3 were significantly decreased whereas the SOD activity and p-Akt expression were significantly increased. Notably, the protections were significantly reversed by pretreatment of wortmannin.

**Conclusion:**

PI3K/Akt pathway activation play a critical role in the protective effects of propofol on intestinal and lung injury induced by ischemia/reperfusion.

## Introduction

 The intestine is probably the most sensitive organ to ischemia/reperfusion (I/R) injury among all the internal organs. Intestinal ischemia/reperfusion (II/R) is a grave clinical event that usually occurs in acute mesenteric ischemia, shock, severe burns, resuscitation, or some surgical operations including intestinal transplantation and abdominal aortic aneurysm surgery^1^. The damages of intestinal mucosal barrier and immune functions of intestine are key factors contributing to injurious consequences which were induced by II/R, and even develop to multiple organ dysfunction syndrome (MODS)^2^. Intestine not only injured itself but also destroyed the remote organs, The lung was the first remote organ affected by II/R^3^. The damage of lung including acute lung injury or even developed to acute respiratory distress syndrome which was the main cause of death in severe patients^4^. 

 Propofol (2,6-di-isopropyl phenol) is a fast, short acting intravenous anesthetic.

 It has the characteristics of rapid recovery, no accumulation after continuous infusion, and is widely used in anesthesia induction, anesthesia maintenance, and sedation of postoperative and ICU patients^5^. Previous studies have indicated that propofol has protective effects on intestinal injury induced by II/R. Moreover, the protection mechanisms were revealed by researchers such as attenuation of intestinal epithelial apoptosis^6^, Inhibiting NADPH Oxidase, effects of antioxidant and in decreasing ET-1 levels^7^, and so on. Recent researches also have shown propofol reduced lung injury induced by II/R, through suppressing oxidative stress and mast cell degranulation, inhibition of lipid oxidation^8^, restraining inflammatory response^9^. However, the exact mechanisms of propofol still have not been sufficiently elucidated in intestinal and lung protections.

 The Phosphoinositide 3-kinase (PI3K)/Akt signaling pathway is an important signaling pathway and widely involved in regulating cell growth and survival, proliferation and apoptosis, glucose metabolism, gene transcription, angiogenesis, inflammation, cell migration and cell cycle . Abundant researchers have reported that PI3K/Akt pathway plays a key role in II/R injury. Sodium/glucose transporter 1 glucose uptake alleviated II/R-induced mucosa barrier dysfunction and bacterial translocation via activation of PI3K/Akt pathway^10^. Sevoflurane inhibits the apoptosis of intestinal mucosal epithelial cells against II/R injury through activation of the PI3K/Akt pathway^11^. The PI3K/Akt pathway also plays an important role in enhancing restitution by HB-EGF against II/R injury^12^.

 Previous studies have indicated that PI3K signaling pathway was involved in the protective effects of propofol on I/R injury. Propofol induced the neuroprotective effect against focal cerebral I/R injury partly due to the activation of PI3K/Akt pathway^13^.

 Thus, the aim of the this study was to investigate whether PI3K/Akt pathway is involved in the protective effects of propofol on intestinal and lung injury induced by II/R; and the role of PI3K/Akt singaling pathway in the mechanism of protective effects afforded by propofol. 

## Methods

 The investigation was performed in accordance with the Guide for the Care and Use of Laboratory Animals published by the U.S. National Institute of Health (NIH Publication No. 85-23, revised 1996) and approved by the Institutional Animal Care and Use Committee of Wuhan University.

 Twenty four healthy Male Sprague-Dawley rats weighing 225 to 275g were purchased from the Department of Laboratory Animal Center of Wuhan University and placed in the standardized environment and free access to food and water under a 12-h light-dark cycles. They were allowed to acclimate to new conditions for a week. All animals were fasted overnight before experiments but allowed free access to water. Twenty four rats were randomly allocated into four groups (n=6 each) which included: Sham, intestinal I/R (II/R), propofol (P), wortmannin (W).

###  Surgical procedure and experimental protocols 

 The model of II/R injury was created as described in previous research^14^. All animals were anesthetized intraperitoneally with an injection of sodium pentobarbital (50 mg/kg). The rats were placed in a supine position and kept the body temperature at 37 ± 1°C by application of a warming blanket set. The left iliac vein was cannulated to administer normal saline as the maintenance fluid. After abdominal shaving and disinfection, the small intestine was exteriorized from abdominal cavity through abdominal midline incision and the superior mesenteric artery (SMA) was exposed carefully. The intestinal ischemia was produced by using atraumatic microvascular clamp to occlude the SMA for 45 min, which was confirmed when the mesenteric artery pulsation ceased and the intestines became blanch immediately. Then the following 2h reperfusion was affirmed by return of intestinal color and mesenteric artery pulsation after clamp removal. Group Sham only performed a midline laparotomy but without suffering I/R injury. Group II/R was subjected to ischemia for 45 min, followed by 2h of reperfusion period. Group P underwent the same surgical procedures as II/R group and administered propofol (20mg/kg/h, Diprivan, propofol 1%, AstraZeneca, Italy) intravenously and continuously at the onset of reperfusion via infusion pumps^15^. Group W was administrated the PI3K inhibitor wortmannin (15ug/kg, Sigma-Aldrich, CA, USA) 25 min before ischemia intravenously, wortmannin dissolved in dimethylsulphoxide and diluted into saline, as previously described^16^; The rest procedures were the same as P group. All animals were sacrificed, and intestines and lungs were harvested and analyzed. 

###  Histologic injury 

 The excised intestinal samples and the lower lobes of the right lungs were fixed in 10% formaldehyde. Tissues were embedded in paraffin wax, then cut into 5 µm sections and stained with hematoxylin and eosin. Finally, histologic injury examined under a light microscope by two pathologists in a blinded manner.

① Assessment of intestinal injury. Chiu’s score was use to evaluate the severity of intestinal injury. In brief, intestinal damage was scored from 0 to 5 as the following criteria: grade 0, normal mucosal structure; grade 1, formation of subepithelial space at the apex of the villus; grade 2, expansion of the subepithelial space with moderate lifting of the Epithelium from the lamina propria; grade 3, massive amount of epithelial lifting down the sides of villi , scattered denuded villous tips may be seen; grade 4, denuded villi with lamina propria, and dilated capillaries exposed; grade 5, presence of hemorrhage, ulceration, and disintegration of lamina propria^17^;
② Assessment of lung injury. The damage of the lung specimens were evaluated by the pathologist on a scale of 0(best) to 3 (worst); evaluation criteria were as follows: grade 0: normal structure of lung tissue; grade 1: slight alveolar wall edema; mesenchyme has small amount of inflammatory cells infiltration; mesenchyme and alveolar cavity appear a little bleeding; grade 2: moderate oedematous thickening of alveolar wall; mesenchyme and alveolar cavity have many inflammatory cells infiltration; capillary congestion, hemorrhage; grade 3: extensive alveolar and interstitial edema; most of the alveoli and mesenchyme have a mass of inflammatory cells infiltration; alveolar bleeding heavily. The mean of the scores represented as lung injury score in all animals^18^.

###  Wet/dry weight ratio 

 AT a distance of approximately 2 cm away from ileocecal valves, 5 cm intestinal tissues were taken and washed in ice-cold normal saline. The specimens were dried with sterile gauze and measured the wet weight, then moved in an oven at 80°C for 24h to obtain the dry weight. The extent of intestinal edema was represented by the wet/dry weight ratio.

 The right middle lobes of the lung tissues were immediately weighed after excising to get the wet weight then desiccated in a drying oven at 80°C for 24h and reweighed to gain the dry weight. The water content of lung was detected by this ratio.

###  Myeloperoxidase activity assay 

 Myeloperoxidase (MPO) activity, an index of the degree of polymorphonuclear neutrophil accumulation, which was measured by spectrophotometer. The intestinal tissues and lung samples were homogenized in cold normal saline using a glass homogenizer. The homogenates were then centrifuged at 1.200g for 10 min. The MPO activities were determined by using MPO assay kits, according to the manufacturer’s instructions (Jiancheng Biologic Project Company, Nanjing, China). The results are expressed as U/g wet tissue.

###  Analysis of malonaldehyde (MDA) level and superoxide dismutase (SOD) activity 

 Intestinal tissues and lung samples were homogenized in cold normal saline and centrifuged. The supernatants obtained and were used for the evaluation of the lipid peroxidation product MDA levels and the SOD activities which were detected according to the manufacturer’s instructions by using chemical detection kits, respectively (Jiancheng Bioengineering Institute, Nanjing, China).

###  Western blotting analysis 

 Intestinal samples and lung tissues were homogenized and centrifuged at 4°C, and the supernatants were collected. The protein concentrations of supernatants of the tissues were measured using the BCA protein assay reagent (Beyotime Institute of Biotechnology, China). Equal amounts of lysate separated on 10% sodium dodecyl sulfate-polyacrylamide gel electrophoresis (SDS-PAGE) and the proteins were transferred onto polyvinylidene difluoride membranes (Millipore, Bedford, MA). Membranes were blocked in 5% nonfat milk at ambient temperature for 1 h, and then incubated with primary monoclonal antibodies against phospho-Akt, caspase-3 (Cell Signaling Technologies, Beverly, MA) overnight at 4°C. The membranes were washed in TBST three times and incubated with the goat anti-rabbit IgG secondary antibody which was labeled by HRP (Jackson ImmunoResearch Laboratories, PA) at room temperature for 1h, and washed again for three times. The Protein bands were visualized by using an enhanced chemiluminescence detection system (Pierce, Rockford, Ill). The densities of the bands were quantified by densitometry through the Quantity One software (Bio-Rad, Hercules, CA).

###  Statistical analysis 

 All data are expressed as the mean±standard deviation. Statistical analysis was performed using SPSS version 13.0 (SPSS, Inc, Chicago, IL, USA). One-way ANOVA (analysis of variance) was used to evaluate the significance among groups, followed by post hoc tests using Fisher’s LSD (least significant difference) multiple comparison test. When *P*<0.05 the results were indicated statistical significance.

## Results

###  Propofol ameliorated intestinal and lung injury induced by II/R 

**Figure 1:**
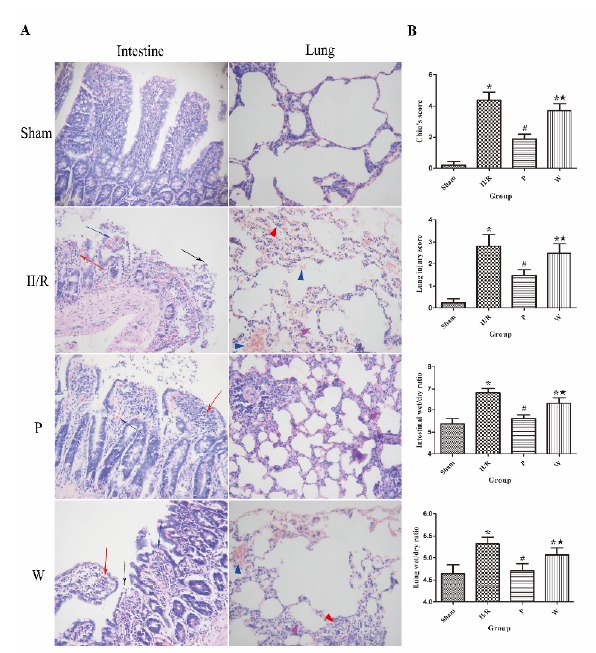
Histologic injury of intestinal mucosa and lung (hematoxylin and eosin staining. magnification ×200) and the evaluation of damages with Chiu’s scores and lung injury scores and wet/dry weight ratios of intestinal and lung tissues. (**A**) Representative images of intestine and lung. In the Sham group mucosal was normal. In the II/R group, significantly severe intestinal injury was seen. Compare with the II/R group, the damage of intestine in the P group was mitigated remarkably. Whereas, in the W group, histologic injury in intestine aggravated compared with the P group. Normal lung tissue was seen in the Sham group. In contrast, seriously lung injury was observed in the II/R group. But compare with the II/R group, the damage of lung was slighter in the P group. However, wortmannin deteriorated the result, alveolar wall thickening, inflammatory cells accumulation, severe alveolar septal congestion, edema and hemorrhage were found in the W group. (**B**) The determination of Chiu’s scores and lung injury scores and wet/dry weight ratios of intestinal and lung tissues. The data are expressed as mean values ± standard deviation (n=6). *P<0.05 *vs.* Sham group. ^#^ *P*<0.05 *vs.* II/R group. ^★^ *P* <0.05 *vs.* P group.

 The histopathology sections were depicted in Figure 1. The morphology in intestinal mucosal was normal in the Sham group. In contrast, the rats in the II/R group showed severe intestinal injury with denuded villus (*black arrow*), significant inflammatory cells infiltration (*red arrow*), exposed capillaries, hemorrhage (*blue arrow*), ulceration, and disintegration of lamina propria. In the P group, the severity of intestinal mucosal injury was mild, there were only capillary congestion (*blue arrow*), slight inflammatory cells infiltration (*red arrow*) and the majority of the intestinal villi architecture was intact. However, compared with the P group, the animals in the W group presented obvious intestinal damage include as follows: massive epithelial lifting, inflammatory cell infiltrates (*red arrow*), denuded villus (*black arrow*), dilated capillaries exposed, slight hemorrhage (*blue arrow*), ulceration may be seen. The Chiu’s scores of four groups were shown in Figure 1. The injury scoring of the II/R and W groups were significantly higher than the Sham group (*P*<0.01).The Chiu’s score in the II/R group was significantly higher than those in the P group (*P*<0.05); dramatically, the protect effect in the P group was reverse by wortmannin (*P*<0.05 *vs*. the P group).

 In the Sham group, the morphology of lung was normal. However, compared with the Sham group, the rats in II/R group showed obviously acute lung injury included that significant thickening of alveolar wall (*purple triangle*), extensive edema and hemorrhage of the alveolar and mesenchyme (*blue triangle*), massive inflammatory cells infiltration (*red triangle*). While the lung pathological damage was attenuated by propofol in the P group; the degree of thickening of alveolar wall and edema were slight (*purple triangle*), congestion of alveolar septal was seen. But, wortmannin reversed the protective effect, aggravated the injury of lung tissue. The lung injury scores of all rats were displayed in Figure 1. The lung injury scoring of the II/R and W groups were significantly higher than the Sham group (*P*<0.01). Compare with the II/R group, lung injury score was significantly lower in the P group (*P*<0.05). Interestingly, Compare with the P group, lung injury score was obviously higher in the W group (*P*<0.05).

###  Effects of propofol and PI3K/Akt signaling inhibition on intestinal and lung wet/dry weight ratio 

 The wet/dry weight ratio was an index of the extent of intestinal and pulmonary edema after II/R. In intestinal tissue, Compared with the sham group, the intestinal wet/dry weight ratios in the II/R group were increased noticeably (*P*<0.05). Compared with the II/R group, the intestinal wet/dry weight ratios in the P group were noticeably decreased (*P*<0.05). Compared with the P group, the intestinal wet/dry weight ratios in the W group were increased noticeably (*P*<0.05). In lung tissue, with the same trend in all groups; compared with the sham group, the lung wet/dry weight ratios in the II/R group were significantly increased (*P*<0.05). Compared with the II/R group, the lung wet/dry weight ratios in the P group were decreased significantly (*P*<0.05). Compared with the P group, the lung wet/dry weight ratios in the W group were significantly increased (*P*<0.05) (Figure 1).

###  Effects of propofol and PI3K/Akt signaling inhibition on MPO activity 

**Figure 2:**
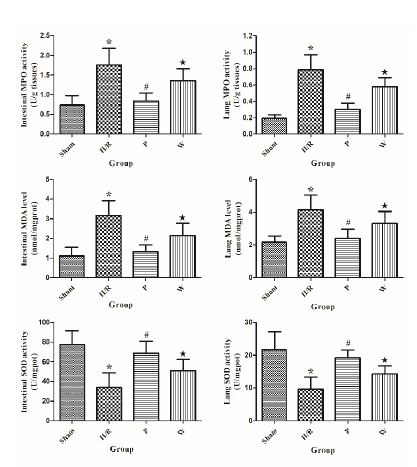
The values of the MPO activities and MDA levels and SOD activities in intestinal and lung tissue of all groups. Data are expressed as mean ± standard deviation (n=6). *P<0.05 *vs.* Sham group. ^#^ *P*<0.05 *vs.* II/R group. ^★^ *P* <0.05 *vs.* P group.

 As shown in Figure 2, MPO is a lysosomal protein which is mainly expressed and released mainly from neutrophils, so the MPO activity is an indicator of the neutrophil infiltration. In intestinal tissue, Compared with the sham group, the intestinal MPO activity was markedly increased in the II/R group (*P*<0.05). Treated with propofol in the P group decreased MPO activity significantly compared with the II/R group (*P*<0.05). Nevertheless, wortmannin hindered the protect effect of propofol, the intestinal MPO activity was increased in the W group compared with the P group (*P*<0.05). In lung tissue, MPO activity in the II/R group was markedly higher than the sham group (*P*<0.05). Compared with the II/R group, MPO activity in the P group was significantly lower (*P*<0.05). MPO activity in the W group was obviously higher than the P group (*P*<0.05).

###  Effects of propofol and PI3K/Akt signaling inhibition on MDA levels and SOD activities 

 The values of the MDA levels and SOD activities of intestinal and lung tissues are shown in Figure 2. In intestinal tissue, compared with the sham group, the intestinal MDA level was markedly increased (*P*<0.05) and SOD activity was significantly decreased in the II/R group (*P*<0.05). Compared with the II/R group, the intestinal MDA level was markedly decreased (*P*<0.05) and SOD activity was significantly increased in the P group (*P*<0.05). Compared with the P group, the intestinal MDA level was obviously increased (*P*<0.05) and SOD activity was significantly decreased in the W group (*P*<0.05). In lung tissue, the lung MDA level was significantly increased (*P*<0.05) and SOD activity was markedly decreased (*P*<0.05) in the II/R group, compared with the sham group. The lung MDA level was markedly decreased (*P*<0.05) and SOD activity was significantly increased in the P group (*P*<0.05), compared with the II/R group. The lung MDA level was obviously increased (*P*<0.05) and SOD activity was significantly decreased in the W group (*P*<0.05), compared with the P group.

###  Effects of propofol and PI3K/Akt signaling inhibition on expression of caspase-3 

**Figure 3:**
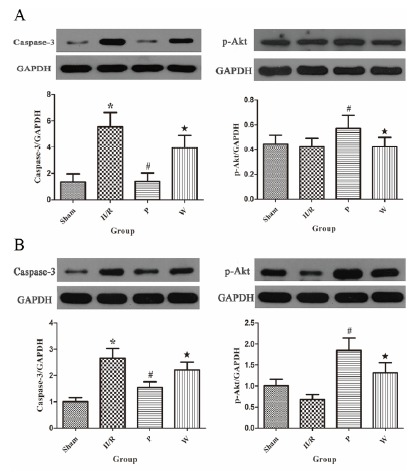
Effects and mechanisms of propofol on the PI3K/Akt pathway in II/R-injured intestines and lungs. (**A**) Representative Western blot showing the expression of caspase-3 and phosphorylation of Akt in intestinal tissues. (**B**) Representative Western blot showing the expression of caspase-3 and phosphorylation of Akt in lung tissues. Both in intestinal and lung tissues, the level of caspase-3 in the P group was significantly decreased compare to the II/R group. The level of caspase-3 in the W group was increased significantly compare to the P group. Moreover, we found that compare with the II/R group, the expressions of p-Akt were markedly increased in the P group; compare with the P group, the expressions of p-Akt were significantly decreased in the W group. Data are expressed as mean ± standard deviation (n=6). *P<0.05 *vs.* Sham group. ^#^ *P*<0.05 *vs.* II/R group. ^★^ *P* <0.05 *vs.* P group.

 The expression of caspase-3 in intestinal and lung tissue was shown in Figure 3. In intestinal tissue, the level of caspase-3 was high in the II/R group. In contrast, the level of caspase-3 in the P group was significantly lower than the II/R group (*P*<0.05). Whereas, the level of caspase-3 in the W group was significantly higher than P group when wortmannin was pretreated (*P*<0.05). In lung tissue, the degree of caspase-3 expression was high in the II/R group. When treated propofol in the P group, the level of caspase-3 was markedly lower compare to the II/R group (*P*<0.05). However, pretreatment with wortmannin reversed the protection of propofol, the level of caspase-3 in the W group was significantly higher than the P group (*P*<0.05).

###  Propofol promotes the phosphorylation of PI3K/Akt signaling after II/R injury 

 As shown in Figure 3. In intestinal tissue, the degree of expression of phosphorylated Akt (p-Akt) was low in the II/R group. When rats suffered II/R and treated with propofol at the onset of reperfusion, the level of p-Akt was significantly increased in the P group (*P*<0.05). Whereas, pretreatment with wortmannin reversed the protection of propofol. The level of p-Akt was significantly decreased in the W group (*P*<0.05). Similarly, the level of p-Akt in the II/R group was low in lung tissue. When animals were given propofol at the onset of reperfusion, the expression of p-Akt was markedly increased in the P group (*P*<0.05). However, pretreatment with wortmannin showed a reversal of the increased expression of p-Akt which was decreased significantly in the W group (*P*<0.05).

## Discussion

 In the present study, we used a rat model which was performed by 45 min occlusion of SMA followed by 2h of reperfusion. II/R caused remarkable intestinal and lung injury which were presented with pathological morphological changes, significant increases in Chiu’s scores and lung injury scores, and noticeably increased wet/dry weight ratios in intestinal and lung tissues, respectively. Above results are in accordance with previous reports^8^ ^,^ ^14^. In comparison, when propofol was treated in rats, the intestinal and lung injuries were significantly attenuated, which is in line with others researches^7^ ^,^ ^8^. Conversely, the protective effects of propofol were reversed by PI3K inhibitor wortmannin.

 Previous researches uncovered that inflammatory response was inevitably involved in the process of organ injury during IR. In our study, inflammation also happened in intestinal and lung tissues, as evidenced by inflammatory cells infiltration under microscope and the increased MPO activities. It is well known that MPO activity is an indicator of neutrophil migration^19^. Accumulated researches suggest that PI3K/Akt signaling pathway plays a critical role in anti-inflammatory response in diverse models and organs (including intestine and lung), and suppression of inflammatory cells accumulation is an important factor^19^ ^,^ ^20^. In recent study, propofol has been reported to have anti-inflammatory effects in various ways. One research reveals that propofol leads to a decrease in plasma TNF-αand IL-6 levels in the model of gut I/R^21^. And another previous study has demonstrated that propofol protects against II/R-induced ALI by suppression of mast cell degranulation^8^. Furthermore, propofol exerts neuroprotection against ischemic brain damage in cerebral ischemia in rats, which relate to attenuation of neutrophil inﬁltration and suppression of inﬂammatory genes^22^. Our study found that the activities of intestinal and lung MPO were significantly increased after II/R, but reduced by treatment with propofol, however, PI3K inhibitor wortmannin reversed this effect and accompanied with the aggravation of tissues damage. Therefore, we speculate that propofol protects intestine and lung from II/R injury due to activation of PI3K/Akt pathway.

 In order to further illuminate whether the activation of PI3K/Akt pathway is involved in the protective effects of propofol on intestinal and lung injury induced by II/R. We tested the MDA levels and SOD activities in intestinal and lung tissues. Oxidative stress is a main factor contributing to II/R injury, and it is also involve in the pathogenesis of ALI induced by IR injury^7^ ^,^ ^8^. Recently, the PI3K/Akt pathway was reported that it’s involved in oxidative stress which induced intestinal and lung tissues damage. Chen *et al*.^23^ indicated that epoxyeicosatrienoic acids alleviate the degree of oxidative stress induced by lung IR injury and this effect is mediated by activation of the PI3K/Akt pathway. Moreover, it has been reported that morphogenic protein epimorphin alleviates oxidative stress injury of intestinal epithelial cells via activation of PI3K/Akt pathway^24^. Intravenous anesthetic propofol and vitamin E are similar in structure, as well as with similar antioxidant capacity. Thus, several studies indicated that propofol has antioxidant property to against oxidative stress of intestinal and lung tissues induced by II/R injury^7^ ^,^ ^8^. Consistent with previous researches^7^ ^,^ ^8^. In current study, intestinal and lung injury were associated with the remarkable decrease of SOD activities which is a major endogenous antioxidant enzyme, and significant increase of the lipid peroxidation product MDA in the II/R group. But treatment with propofol increased SOD activities and decreased MDA levels that was associated with the reduced Chiu’s scores and lung injury scores. Interestingly, pretreatment with wortmannin significantly decreased the SOD activities of intestinal and lung tissues and increased their MDA levels, indicating that propofol exerts protection against oxidative stress via activation of the PI3K/Akt pathway.

 Accumulated studies have reported that apoptosis is an important mode of cell death and plays a critical role in the pathophysiologic process of I/R injury^23^. Apoptosis involves a series of complex cellular events, and caspases, one of proteolytic enzymes, initiate cellular damage finally. As far as we know, pro-apoptotic factor caspase-3 is the one that most relates to apoptosis among the various caspases^25^. So we choose to detect caspase-3 in tissues in current study. It is well known that the PI3K/Akt pathway plays a pivotal role in promoting cell survival by inhibition of apoptotic pathways; moreover, this signaling pathway regulates apoptosis in many ways, such as targeting of Bcl-2 family proteins, GSK-3 (glycogen synthase kinase 3), forkhead transcription factors and regulation of protein synthesis, p53, NF-κB^26^.

 Recent reports indicated that PI3K/Akt pathway has the ability to reduce IR injury through the antiapoptotic property. We give some examples as follows. A sodium/glucose transporter 1 uptake reduces epithelial cell apoptosis and barrier damage by activation of PI3K/Akt pathway^10^. Additionally, cytochrome P450 epoxygenase 2J2 markedly attenuated cell apoptosis in lung tissues induced by IR via activation of PI3K/Akt pathway^23^.Many lines of evidence have shown that propofol has antiapoptotic effect in various tissues. Recent research reported that propofol exerts neuroprotective effect through inhibiting neuronal apoptosis via activating the PI3K/Akt pathway after focal cerebral IR injury^13^. Propofol can also attenuate intestinal damage by inhibition of intestinal epithelial apoptosis after both IR injury and burn injury^6^. Our present results showed that II/R significantly increased the expression of caspase-3. However, treatment with anesthetic propofol remarkably activated Akt and decreased expression of caspase-3. The PI3K inhibitor wortmannin inhibited the phosphorylation of Akt and increased expression of caspase-3 and reversed protection induced by treatment with anesthetic propofol. All together, these results indicate that the PI3K/Akt pathway was involved in the protection of propofol against intestinal and lung injury induced by II/R through inhibiting cell apoptosis.

 In addition, Ng *et al*.^27^ suggest that oxidative stress induced by pulmonary IR injury activates the mitochondrial pathway which is known as the intrinsic pathway of apoptosis, and then results in lung apoptosis. Recently, a study indicated that reactive oxygen species (ROS) promote cell apoptosis processes mediated by the sphingomyelinase-ceramide pathway in various tissues^6^. Moreover, Baregamian *et al*.^28^ revealed that mitochondria are the main source of intestinal apoptotic signaling during oxidative stress.The reason is that excessive ROS damaged mitochondria, triggering apoptotic process via overexpression of pro-apoptotic proteins and release of cytochrome c^23^ ^,^ ^29^. A research reported that propofol attenuates intestinal epithelial apoptosis following II/R, which may be owed to its antioxidant property^6^. Another research indicated that epoxyeicosatrienoic acids inhibit ROS production via activation of PI3K/Akt pathway after lung IR, which mitigates mitochondria dysfunction, at the end, cell apoptosis processes is blocked^23^. Unfortunately, we haven’t studied the relationship between antioxidant and antiapoptotic properties of propofol. Meanwhile, it remains unclear whether the antiapoptotic effect of propofol on lung cells is related to antioxidant property of propofol in current study, which need to be further investigated.

 We noticed that wortmannin reversed only partly the protective effects of propofol. Besides PI3K/Akt pathway, other protective mechanisms may exist. Recent study suggest that mitogen-activated protein kinase/extracellular signal-regulated kinase 1/2 (MEK/ERK1/2) and PI3K/Akt are both activated by heparin-binding epidermal growth factor-like growth factor and enhance restitution during intestinal IR^12^. In addition, The Janus kinase/signal transducer and activator of transcription (JAK/STAT) pathway is participate in I/R-induced intestinal injury; and ischemic postconditioning relieves the II/R injury through inhibiting JAK/STAT pathway^30^.

 There are several limitations in our study. First, we used only the inhibitor wortmannin to investigate the role of PI3K/Akt signaling pathway in current study. Accordingly, the PI3K or Akt genes knockout models or the RNA interference technology should be used in further research, which will be in favor of proving their specific roles in our study. Second, we tested only MPO activity, but did not detected the expression of inflammatory factors. This may not be fully reflected the extent of the inflammatory response. Third, besides caspase-3, we should detect apoptosis-related genes and other indicators to provide stronger evidences and determine the existence and degree of apoptosis.

## Conclusions

 Propofol protects intestine and lung from II/R injury through inhibiting cell apoptosis, oxidative stress and neutrophil accumulation. We demonstrated that the protective effectives of propofol were mediated partly by PI3K/Akt pathway. It may be provided a theoretical basis for the molecular mechanisms of the protective effects produced by propofol; and the molecular mechanisms need to be explored further.
